# Toward Inclusive Design Heuristics for Digital Health Interventions for the Aging Population: Scoping Review

**DOI:** 10.2196/79449

**Published:** 2025-12-22

**Authors:** Kerstin Denecke, Lana Cvijic, Carolyn Petersen

**Affiliations:** 1 Department Engineering and Computer Science Bern University of Applied Sciences Biel Switzerland; 2 Department of Practice Administration Mayo Clinic Rochester, MN United States

**Keywords:** older adults, population aging, health challenges, social challenges, disabilities, impairments, visual impairment, hearing impairment, socio-cultural diversity, linguistic diversity, scoping review, inclusive design, inclusive design heuristics, inclusive design recommendations, inclusive design guideline, digital health solution, digital health intervention, human-centered design, co-creation, mobile phone

## Abstract

**Background:**

Digital health interventions (DHI) deliver health-related services in a digital manner. Meant for older adults, they must be tailored to address their needs. This may be by applying inclusive design principles. Inclusive design is an approach that aims to accommodate the needs of a broad spectrum of users, taking into account health-related factors, socioeconomic status, age, cultural background, language diversity, and other factors.

**Objective:**

This review aims to collect best practices on the inclusive design of DHIs for older adults and aggregate them into a set of design guidelines.

**Methods:**

We examined peer-reviewed papers from 3 databases that described a design and development process of a DHI specifically designed for users aged 60 years or older, and used inclusivity to design the solution. The process followed PRISMA-ScR (Preferred Reporting Items for Systematic Reviews and Meta-Analyses extension for Scoping Reviews) guideline and PRISMA-S (Preferred Reporting Items for Systematic Reviews and Meta-Analyses literature search extension) checklist. Information on the DHIs and their design process, as well as facilitators and barriers for adopting DHIs by older adults, was extracted.

**Results:**

Of 1276 records, 40 papers were included and considered for data synthesis. DHIs are provided through a broad range of technical platforms (mobile apps [20/40], web-based platforms and web apps [6/40], voice and virtual assistant technology [2/40], telehealth and remote monitoring systems [4/40], and tablet-based and specialized systems [8/40]). Sometimes, their design process included older adults (2/40) but also clinicians (1/40), designers and developers (3/40), and researchers (3/40), as well as other community stakeholders (3/40). The derived design heuristics to be considered for inclusive design comprise 11 aspects covering multiple dimensions: visual design and readability, navigation, accessibility, customization and personalization, social engagement and support, learnability, multiplatforms and device compatibility, motivation, feedback and user engagement, security and privacy, inclusive language, and costs. Barriers range from age-related health issues to technical hurdles related to access or connectivity.

**Conclusions:**

The inclusive design of DHIs for older adults extends beyond usability and user interface design. This study highlights the critical role of co-designed DHIs in addressing persistent challenges of isolation, limited mobility, and access to care among older adults. Older adults must be placed at the center of development, with their needs and challenges identified and addressed in the solution. By enabling tailored, locally relevant digital experiences, co-design empowers older adults to engage meaningfully with health services despite infrastructural and socioeconomic barriers. The list of inclusive design aspects and recommendations provides a starting point for DHI developers to create functionality that supports many needs and goals of older adults. Future work must validate our results from a practical perspective. The adoption of our heuristics in practice could be fostered by developing concrete methods that implement them.

## Introduction

### Background

Worldwide, the population of people aged 60 years and older is growing at a faster rate than any other age group [1]. This shift is referred to as population aging, a demographic phenomenon characterized by an increasing number of people aged 60 years and older, followed by a simultaneous decline in the younger population [2]. It is driven by increased life expectancy, with the global population aged 60 years or older expected to reach 2.1 billion by 2050 [3,4]. Although this initially emerged in high-income countries, it has now become an issue in many developing countries as well [2]. As people age, they face a range of health-related and social challenges. Older adults’ quality of life is strongly influenced by access to health care, social inclusion, availability of caregivers, and opportunities for meaningful engagement in communities they are part of [5]. Additionally, older adults are at a higher risk of experiencing different health conditions, which include cognitive impairment, chronic pain, and noncommunicable diseases such as cardiovascular diseases, diabetes mellitus, and cancer [3,6]. Therefore, the promotion of health and well-being among older adults becomes a priority for achieving healthy aging [7]. Addressing the health needs of the aging population is a pressing public health priority, especially given the increasing health care costs associated with aging [8].

The focus of this work is on digital health interventions (DHIs), which refer to the use of digital technologies to deliver health-related services, information, or support. The main aim of DHIs is to improve health outcomes, health care delivery, and health system efficiency. DHIs present innovative, cost-effective solutions to address the health-related and social challenges older adults face. DHIs have the potential to empower older adults to actively engage in their health management by promoting early detection of conditions, prevention, and personalized care [9]. The World Health Organization classifies DHIs into various categories based on their role within the health care system [10]. One category is the DHIs for personal health tracking, which includes mobile health apps (mHealth apps), phone-based sensors, wearables, and web-based platforms. These DHIs enable users to monitor their vital signs, levels of physical activity, medication adherence, and other health-related data. Emerging evidence indicates that older adults are becoming increasingly interested in digital technologies, and DHIs can be effective for them [11]. However, the success of these interventions depends on their accessibility and usability, which must be tailored to meet the needs of older adults. This can be achieved by the implementation of an inclusive design approach [12].

Inclusive design is an approach that aims to accommodate the needs of a broad spectrum of users, considering factors such as socioeconomic status, gender, age, ethnicity, and language diversity [13]. This approach is particularly important when designing for older adults, as there is no “typical” older person. Aging is an individualized process, which is shaped by both physical and social environments that influence individuals’ health behaviors and needs [4]. Consequently, DHIs must be inclusively designed to address the wide range of needs of this heterogeneous user group. DHIs that do not take into account the unique needs and abilities of older adults are likely to fail in their ability to support use and adoption by a large and growing segment of the population [14].

### Previous Work

Several checklists and guidelines have already been developed in the field of inclusive design for DHIs targeting older adults. Much of the existing research has concentrated on mHealth apps [15-17]. For instance, Paez et al [17] investigated the usability challenges older adults face when using mobile apps and formulated a primary set of recommendations based on their findings. Similarly, Liu et al [16] proposed a set of recommendations and identified five persuasive design elements that enhance interfaces for older adult users, which include reminders, social features, gamification, personalized interventions, and health education. However, their focus remained limited to visual aspects of mobile interfaces, excluding broader DHI categories and inclusive design aspects. Gomez-Hernandez et al [15] used a scoping review methodology and proposed a guideline for the design of mobile apps for older adults that do not specifically target health. One checklist was developed in the Got-IT (Guidelines on Target Assessment for Innovative Therapeutics) project, which aimed to assist the design of inclusive digital health solutions targeting the promotion of healthy lifestyles among older adults [18], also mostly focusing on the presence and design of usable interfaces. Innovative work in this field was conducted by Boot et al [14], who aimed to understand the full range of needs of older adults and proposed comprehensive design recommendations, published in book format. Furthermore, Brewer [19] assessed the accessibility of nonvisual technologies, thereby advancing the field of inclusive design for individuals with visual impairments.

In contrast to previous contributions in this area, we aim to explore a broader range of DHIs for personal health tracking that target different health domains and address different types of age-related impairments, such as cognitive, hearing, and visual impairments. We focus on DHIs that have been co-designed with a target user group and evaluated to reflect design elements that are well perceived by older adults. Furthermore, our review considers nonvisual DHIs, such as voice assistants, which are particularly relevant for older adults with visual impairments. In addition, we aim to examine DHIs from different parts of the world to consider the social, economic, linguistic, and cultural aspects that play an important role in inclusive design.

### Objectives

This scoping review is part of a 3-phase development process of inclusive design heuristics of DHIs for older adults. The entire process is based on the methodology for developing usability and UX (user experience) heuristics proposed by Quiñones et al [20], which consists of (1) a knowledge generation phase, (2) formulation of heuristics, and (3) an iterative validation and improvement of heuristics. We conducted the scoping review along with expert interviews and a workshop with older adults during the knowledge generation phase to gather the knowledge necessary for developing the heuristics. This paper focuses on the scoping review of the design and development process of DHIs that specifically target older adult users. From studies, we aimed to gain insights that inform the inclusive design of DHIs, considering the diverse requirements and needs of older adults. Finally, we aggregated the collected information and proposed an initial set of design heuristics. We define heuristics as the design aspects (eg, interface design, accessibility, and personalization) and recommended design elements (eg, providing an audio content option for users with visual impairment), which promote the inclusive design of DHIs. We defined the following research questions to guide this review: (1) What are the key principles of inclusive design applied to DHIs for older adults? (2) How do current DHIs address the specific needs of older adults, including cognitive, sensory, and motor impairments? (3) What heuristics have been proposed in the literature for designing user-friendly DHIs for older adults? and (4) What best practices for inclusive design have been validated or proven effective for DHIs targeting older adults?

## Methods

### Overview

Given the complexity of the topic, a scoping review methodology was selected to systematically explore relevant papers that described the design of the DHIs for older adults [21]. The inclusion and exclusion criteria for selecting the papers are summarized in Textbox 1. PRISMA-ScR (Preferred Reporting Items for Systematic Reviews and Meta-Analyses extension for Scoping Reviews) guideline [22] and PRISMA-S (Preferred Reporting Items for Systematic Reviews and Meta-Analyses literature search extension) checklist [23] were followed to ensure methodological rigor and accuracy of the results. The completed PRISMA-ScR checklist is provided in Multimedia Appendix 1. The PRISMA-S checklist can be found in Multimedia Appendix 2. The review methodology was not registered in advance.

**Overview of the criteria used to include and exclude papers in our scoping review.**

**Inclusion criteria**

- Study types: peer-reviewed research papers.
- Intervention: digital health interventions designed specifically for older adult users.
- Population: older adults aged 60 years and older.
- Language: English or German.

**Exclusion criteria**

- Study types: conference abstracts, study protocols, (systematic) reviews, editorials, letters to the editor, errata, corrigenda, corrections, comments, retracted papers, responses, conference proceedings, books, and book chapters.
- Intervention: digital health intervention is not described, a lack of information on the design and development process.
- Population: people younger than 60 years.
- Language: all other languages.

### Search Strategy

We searched three relevant databases for this topic: IEEE Xplore, Scopus, and PubMed. All database searches were initially conducted on January 12, 2025, and were rerun on September 22, 2025, using the same search strategy to identify newly published studies. Each database was searched via its web interface, and searches were run separately in each database rather than simultaneously on a single platform. No study registers were searched. No additional online sources (eg, websites, conference proceedings, and journal tables of contents) were searched to identify eligible studies, and citation searching was not undertaken for the additional study selection. We did not seek additional sources by contacting researchers, nor did we use any other search methods beyond those described above to identify further relevant studies for this scoping review. From our research questions, we retrieved the three keywords: “DHI,” “older adults,” and “inclusive.” These were used to develop the search string. The final search string was as follows: “(“digital health” OR “eHealth” OR “mHealth” OR “telemedicine” OR “telehealth” OR “digital healthcare tools” OR “digital therapeutics” OR “mobile health apps” OR “online health platforms” OR “wearables”) AND (“aging population” OR “older adults” OR “seniors” OR “aged individuals” OR “older persons” OR “geriatric population” OR “aging individuals” OR “aging adults” OR “senior citizens” OR “elderly population”) AND (“inclusive” OR “accessible” OR “equitable” OR “universal design” OR “user-friendly” OR “culturally sensitive” OR “inclusive design” OR “barrier-free” OR “non-biased” OR “non-discriminatory” OR “diverse” OR “adaptive” OR “tailored for all”).” To keep the search broad, this search strategy was applied to the title and abstract fields in each database. We did not use any previously published or validated search filters. All search strategies were developed specifically for this scoping review and were not formally peer reviewed by an independent expert before execution. The reproducible search strategies for all databases are provided in Multimedia Appendix 3.

### Study Selection

A total of 1284 papers were found. Duplicate papers (n=508) were removed before the screening process. Papers (n=1276) were screened independently by authors (KD, LC, and CP) who examined the title and abstract of the papers to determine if they met the initial eligibility criteria (Textbox 1). The screening was carried out in the Rayyan software to facilitate the screening and deduplication process [24]. We did not perform a critical appraisal of the included sources of evidence because many studies used experience-based methods that do not fit within the critical appraisal checklist, such as prototype usability research, digital tool use evaluation, and similar experience-based approaches.

### Data Extraction

Before the analysis of the papers, all authors were given one paper to perform the initial extraction and to agree on the data extraction items and eligibility criteria. In addition, a quality assurance meeting was organized by the three authors (KD, LC, and CP) who performed the data extraction to agree on the data items to be extracted and to check that the items were understood in the same way by all authors. The final list of data items that were extracted is documented in Textbox 2. All data collected were documented in a data extraction spreadsheet.

**Definitions of aspects that were extracted from the included papers during the data extraction phase.**

- “Age group” involved in the study: only when participants older than 60 years of age were involved in the design process was it considered eligible for further analysis.
- “Disease or condition” concerns whether the digital health intervention (DHI) was designed with a specific disease or condition in mind.
- “Disability”: to achieve inclusive design, different impairments should be considered to create an accessible and inclusive DHI. For this reason, we looked for specific disabilities that a DHI targeted to see if there are design practices for different impairments (eg, visual or hearing impairment).
- “DHI developed during the study” refers to the name and type of the DHI.
- “Method used to involve older adults” in the design process of the DHI (eg, qualitative interview and focus group).
- “Was a study on the DHI conducted?” This item was answered with a yes or no option.
- “Parties involved in the study on DHI” refers to the research subjects that were involved in the study.
- “Parties involved in the development process of DHI” refer to the research subjects that were involved in the development process.
- “Definition of inclusive design”: when the term “inclusive design” was defined, this definition was extracted.
- “Frameworks or guidelines” applied in the study: all types of frameworks were extracted (including health and behavioral science frameworks), and not only the design frameworks or guidelines.
- “Design principles or heuristics applied to achieve inclusivity”: we extracted both the design elements applied to the DHI and the improvement recommendations and suggestions for design mentioned by older adult participants in the study, which would make DHIs more inclusive from their point of view.
- “Barriers” to adoption of DHIs among older adults refer to aspects that hinder the adoption of DHIs by older adults.
- “Facilitators” to the adoption of DHIs among older adults refer to aspects that facilitate the adoption of DHIs by older adults.

### Synthesis of Data

Following the PRISMA (Preferred Reporting Items for Systematic Reviews and Meta-Analyses) guidelines, we performed a narrative synthesis of the results and presented the results in a descriptive manner or in tables. The extracted free text was aggregated by a thematic analysis conducted by one author (KD). She reviewed all entries for one item and identified common themes. Additionally, a locally run language model (Llama3; Meta) was used to confirm the identified themes. The prompt used was: “Conduct a thematic analysis on the following information extracted on [item]. Provide the identified themes.” A second author (LC) checked the aggregated themes for completeness and correctness.

## Results

### Overview

**Figure 1:**
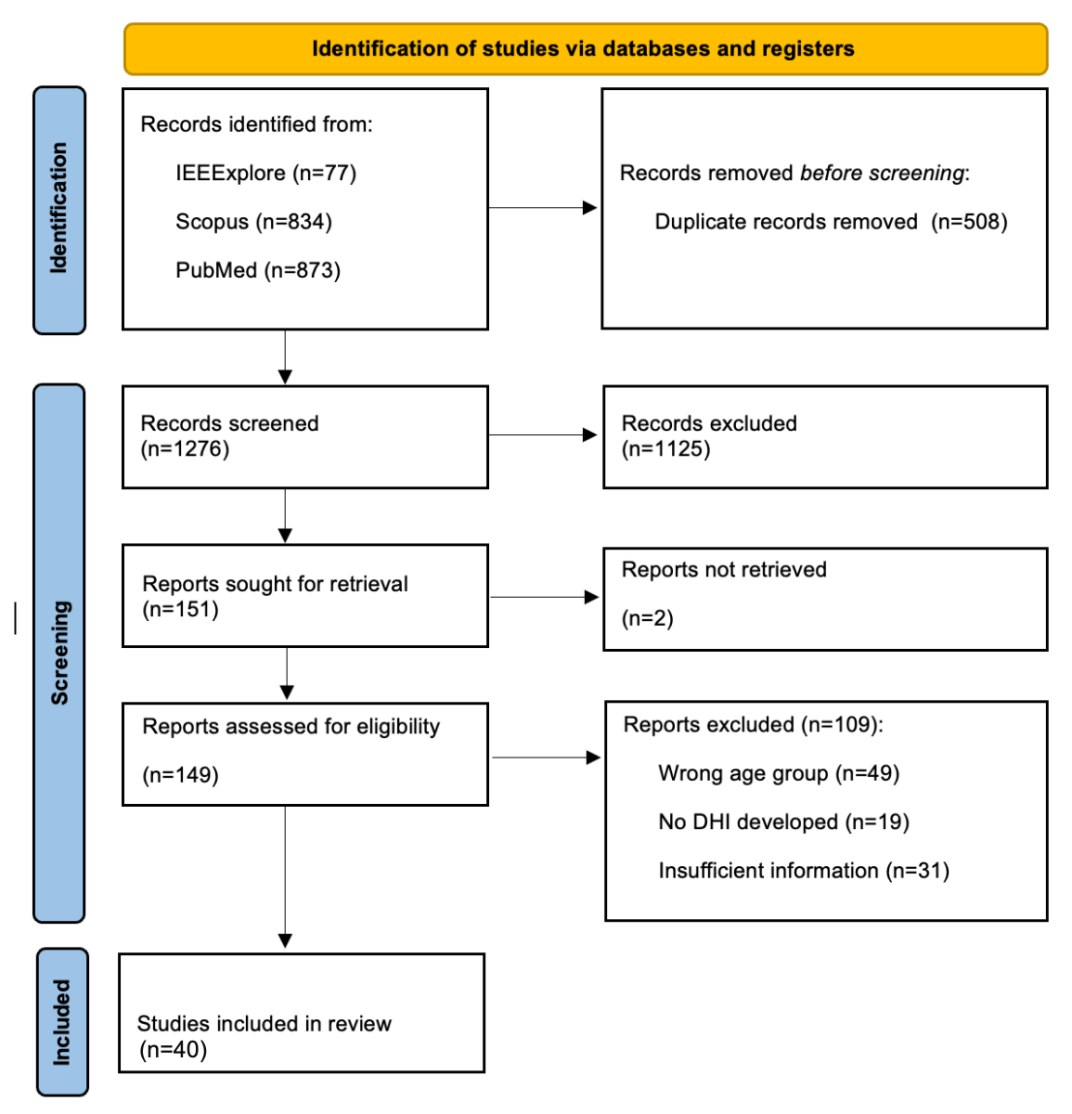
PRISMA flowchart for study selection. DHI: digital health intervention; PRISMA: Preferred Reporting Items for Systematic Reviews and Meta-Analyses.

The literature search yielded 1276 unique records that were screened at the title and abstract phase. A total of 149 papers were screened at the full-text stage, and a total of 40 papers were included and considered for information extraction (Figure 1). Papers were excluded because of the wrong age group, insufficient information on the design process and inclusive design elements of the DHI, or the wrong type of publication. An overview of the most important insights from the extracted studies is shown in Table 1. In this table summary, we included the studies that described the applied design elements aimed at making the DHI more inclusive for older adult users. Details on the extracted data are summarized in the next sections. The detailed data extraction spreadsheet and information on the exclusion reasons are provided in Multimedia Appendix 4.

**Table 1:** Main findings during the data extraction process. This table presents the age groups considered by each DHIa, the health-related domains addressed, the type of DHI, and the design elements implemented to promote inclusive design. Only studies (30/40) that report design elements are listed.

First author and reference	Age group (years)	Health-related domain	Type of DHI	Applied design elements to achieve an inclusive design of the DHI
Beukema et al [25]	65+	Frailty	Online platform for screening result messages	Personalized results messages Personal messages for communication of negative results Standard messages for communication of positive results Empathic communication for high-impact diseases
Dhillon et al [26]	60+	Prevention	Telehealth platform for the prevention of health-related conditions	Large fonts Linear navigation structure Icon-based menu Personalization options (eg, users choose the apps according to their preferences) Social element and functionality for networking to provide social and emotional support Multidevice compatibility
Jansons et al [27]	60+	N/A^b^	Telehealth platform for rehabilitation	Voice-based interaction Personalized exercise programs Personalized reminder functionality Educational material about the exercises and their benefits
Orellano-Colón et al [28]	65+	Functional disabilities	Web-app to support the independently living older adults with physical impairment	No password or username required No installation needed Multidevice compatibility Intuitive navigation High-contrast colors Large-sized buttons Limited options and information on each page Visual elements (images and videos featuring real older adults)
Böttinger et al [29]	65+	N/A	Self-management app for early risk detection	Large font sizes Visual elements (symbols, icons, and images) Instructional videos for the onboarding in the DHI Voice-based assistant Personalized reminders with sounds Social element (eg, possibility to share experiences and contact other users) Personalization options (eg, personalized recommendations to support well-being and personalizable features) Costs of the DHI are covered by the insurance companies Evidence-based content
Ariza-Vega et al [30]	65+	Hip fracture	mHealth app to improve physical activity	Simple UIc design Large font-size Multimodal content (voice-over and audio-visual content) Personalized exercise programs and exercise instructions Comprehensive educational content
Mansson et al [31]	70+	Fall prevention	mHealth self-test app for balance and leg strength	Clear instructions and onboarding Simple UI design Multimodal content (written and video material) Adaptability for different levels of cognition Foster the credibility of the DHI and feel safe with it Recognize the added value for the user
Wegener et al [32]	65+	Chronic obstructive pulmonary disease	Multiplatform conversational agent to facilitate daily communication	Co-design with target users Personalized features, content, and frequency of messages Clear onboarding Multimodal content (written and video material) Possibility to share data with caregivers and health care professionals Educational content Text-based and voice-based reminders to foster motivation Activity and progress tracking option Pop-up reminders and calendar function for social activities
Strandberg et al [33]	65+	Chronic illnesses	Video-based tool for self-management at home	Cocreation with target users Zoom functionality Instructions and educational content Large font-size and high contrast
Sobrinho et al [34]	60+	N/A	mHealth app for promotion of health habits	Adjustable font-sizes Intuitive UI Vivid colors Compatibility with screen-readers Self-explanatory icons Feedback after task completion for motivation Easy registration process with reduced barriers
Mazuz et al [35]	60+	Fall prevention	mHealth app for fall prevention	Personalized reminders to foster motivation Self-reporting diary functionality Video explanation of exercises
Ghorayeb et al [36]	65+	N/A	Smart-home interface to enable health and self-monitoring	Co-design with different cultural probes Personalized recommendations Reminders (date, time, weather, appointments, and location of the wearable) Visualized data for better understanding and an option to choose who can see their data Home monitoring functionality for safety
Khamaj and Ali [37]	60+	Kidney failure	mHealth app for emergency services	User-centered design and feedback collection throughout the design process Intuitive navigation Optimized button placements Adjustable font sizes and high contrast for better readability Large touch targets Multidevice compatibility Screen-reader compatibility SOS^d^ message feature and quick dial options for emergency services
Wikberg-Nilsson et al [38]	71+	Sensory decline	Tablet-based app to promote healthy aging	Large-sized text Simple visual design elements Use of visual elements to complement the text-based content (eg, pictures and symbols) Conscious use of pictures to avoid the stigmatization of older adults Simple UI Clear and easy navigation Personalization options (eg, for images, content, and text fonts)
Teh et al [39]	60+	N/A	mHealth app with an avatar that serves as a coach for healthy aging	Use of gamification elements to foster motivation Compatibility with common browsers Content in multiple languages Personalized content and recommendations (eg, nutrition recommendations based on the cultural and societal context) Multimodal content (text-based or audio-based)
Moral et al [40]	70+	Frailty	mHealth app for frailty follow-up	Cocreation with target users Clear and simple UI design Large buttons Large-sized text Multimodal content Use of visual elements to complement the text Educational content
Athilingam et al [41]	Average 63	Heart failure	mHealth app for self-care management	Personalized reminders and alerts (eg, for the medication) Evidence-based and trustworthy educational material Graphic representation of vital signs
Adami et al [42]	65+	Age-related conditions	System for health monitoring and well-being	Voice-based interaction Visual elements to complement text-based content Simple UI design Well-structured navigation
Daniels et al [43]	65+	N/A	mHealth app for promotion of physical activity	Customization options for improved user experience Daily reminders Tailored feedback Messages tailored to users’ specific needs and preferences Accurate and easily understandable information Multimodal content (videos featuring older adults) Social element (eg, exercising with peers) Easy to use navigation Clear fonts Large buttons
Thaduangta et al [44]	60+	N/A	Smart health care system for biomedical data collection	Large and adjustable font sizes No flash content Clear instructions Single mouse clicks High contrast Clear navigation bar Way to prevent errors Demonstration of results in a simple and clear way SOS button functionality
Murabito et al [45]	64+	N/A	mHealth app for health data collection	Clear and simple instructions Gamification elements to boost engagement Large fonts User-friendly colors Personalization to satisfy different user needs No one-size-fits-all solution
Hawley-Hague et al [46]	60+	N/A	mHealth app for rehabilitation exercises	Simple UI design High contrast Large font-sizes
Park et al [47]	Average 68.1	Cognitive impairment	Telemedicine-based exercise program	Intuitive UI Minimized required manual interactions Instructions in video, audio, and text-based format
Hunter et al [48]	70+	Chronic conditions	Telehealth system with smart-home sensors	Customization of the DHI according to the users’ unique routine
Revenäs et al [49]	65+	Physical activity	mHealth app for promotion of physical activity	Simple navigation Good readability Precise and meaningful texts Large font-sizes Visual material (eg, videos for demonstration of exercises) Touch pens for users who have difficulties with touch screens Tutorial with the clarification of the app structure and page content
Liu and Su [50]	60+	N/A	Augmented reality–based system that provides support in hospital visits	Multimodal content (voice-based interaction and visual cues) Clearly explained information and trustworthy content, validated by experts Nurse-like virtual agent with gentle facial expressions, calm tone, and warm body language to foster trust and approachability Color scheme that supports readability Personalized experience (eg, personalized pacing and option to make the interaction with the agent slower)
Hultman et al [51]	60-75	Sedentary time	Self-management app for the reduction of sedentary time	Adaptable design of the DHI (eg, technically advanced users have the possibility to add more features to the app) Multidevice compatibility Multimodal content (audio, text, and video) Onboarding guidance with explanations of functionalities Clean layout Easy-to-understand language, simplified text, and information Social element (eg, the app provides information on social events) Reminders to boost adherence to new habits Gamification elements (eg, rewards) to foster motivation
Daniels et al [52]	65+	Physical activity	mHealth app for promotion of physical activity	Educational content (eg, about mental health, retirement, and nutrition) Checkbox to track the articles read Social element (eg, community calendar option and option to meet the exercise partners) Multimodal content (text, video, and audio) Personalization (eg, an onboarding questionnaire to personalize content) Simple layout, bright colors, large icons, and large font-sizes
Chopvitayakun et al [53]	60+	Nutrition	mHealth app for nutrition calculation	High contrast colors Large font-sizes Minimal onboarding process to reduce cognitive overload Visual content (eg, cons and labels, and pie charts)
Bao et al [54]	60-85	Physical and mental well-being	System for managing health and mobility (including wearable sensors, mHealth app, and cloud-based data storage)	Gamification elements to boost motivation Culturally adapted content (eg, icons to represent macronutrients, using hand-size references for portion estimation, using predictive text entries with culturally adapted dish names) Bilingual interface Adjustment of colors based on the current lighting to ensure constant readability Help button that helps with the navigation

^a^DHI: digital health intervention.

^b^N/A: not applicable.

^c^UI: user interface.

^d^SOS: Save our Souls.

As no critical appraisal was performed, no quality ratings of the included sources of evidence are presented.

### Main Characteristics of the Included Papers

The scoping review identified 40 studies published between 2013 and 2025 that addressed the inclusive design of DHIs for older adults. The temporal distribution demonstrates a marked increase in recent years, with 18 (45%) studies published in 2024-2025 alone, reflecting growing research interest in this domain. The publication trend shows modest activity from 2013-2022 (1-4 studies per year), followed by acceleration from 2023 onward (6-12 studies per year).

The included studies predominantly targeted older adults aged 60+ and 65+ years, with 23 different age group specifications identified across the corpus. Two papers [27,28] mentioned only an average age instead of an age range.

The DHIs addressed some specific medical conditions or aimed to address age-specific conditions and prevention of age-specific conditions in general: frailty [25,40], prevention [26], hip fracture, fall prevention [31,35], chronic obstructive pulmonary disease [32,55], chronic illness [33,48], mild cognitive impairment [47,56], kidney failure [37], heart failure [41], age-related conditions [42], underactivity [57], melanoma [48], and depression [58]. Eighteen papers did not mention any medical condition or symptoms on which the solution centered. Partially, they mentioned health aspects they focused on, such as physical activity. The following specific disabilities were considered in the design of the solutions by 6 papers: Functional disabilities [28], cognitive impairment [47,56], sensory decline [38], blindness or low vision [59], or hearing impairment [60]. Overall, 34/40 (85%) papers reported a study with their DHI ranging from a requirements collection study to qualitative and quantitative cocreational studies, to a feasibility study, and clinical trials.

Only 3 papers defined inclusive design. Khamaj et al [37] define inclusive design in a way that “users’ physical capabilities are considered, and the software is made accessible and flexible to accommodate a variety of mobility or dexterity challenges, especially those that affect older persons.” Wikberg-Nilsson et al [38] refer to a publication and define inclusive design as “an approach not comprising a set of fixed criteria but emphasizing the design practice responsibility of embracing users with various special needs [61,62].” Chopvitayakun et al [53] use the term “user sensitive inclusive design,” which refers to the expansion of user-centered design principles that explicitly address the diverse requirements of older adult individuals and those with disabilities [63].

### DHIs and Their Development Process

The DHIs reported in the papers can be grouped into 5 categories as shown in Textbox 3. Mobile apps (ie, installed apps) included apps for supporting health promotion and education (eg, a physical activity promotion app) [37,43,49,52,54,57,64,65], for self-care and chronic condition management (eg, an app for frailty follow-up) [34,40,41,60], for rehabilitation and recovery (eg, an app for rehabilitation exercises) [30,46], for balance and physical functioning testing (eg, a self-test for leg strength and balance) [31], for self-management [35,50,55], and for nutrition calculation [53]. Web-based platforms and web apps are DHIs accessed via a web browser. Symptom checkers [66], conversational agents [32], and a self-screening and monitoring platform [29] were provided as a web-based platform, to mention a few examples. Voice and virtual assistant technology tools use speech and artificial intelligence interfaces to assist in remote care. Telehealth and remote monitoring systems include smart home sensors with telehealth [48] or smart health care systems for biomedical data collection [44]. Tablet-based and specialized systems include a video feedback tool [33], a telemedicine-based exercise program [47], tablet-based drawing and dragging tasks [56], a sensor-based system for monitoring and management of health and mobility [54], and an augmented reality–based system that provides support for older adults in hospital visits [50]. Use of wearable technologies was not identified.

**Figure 2:**
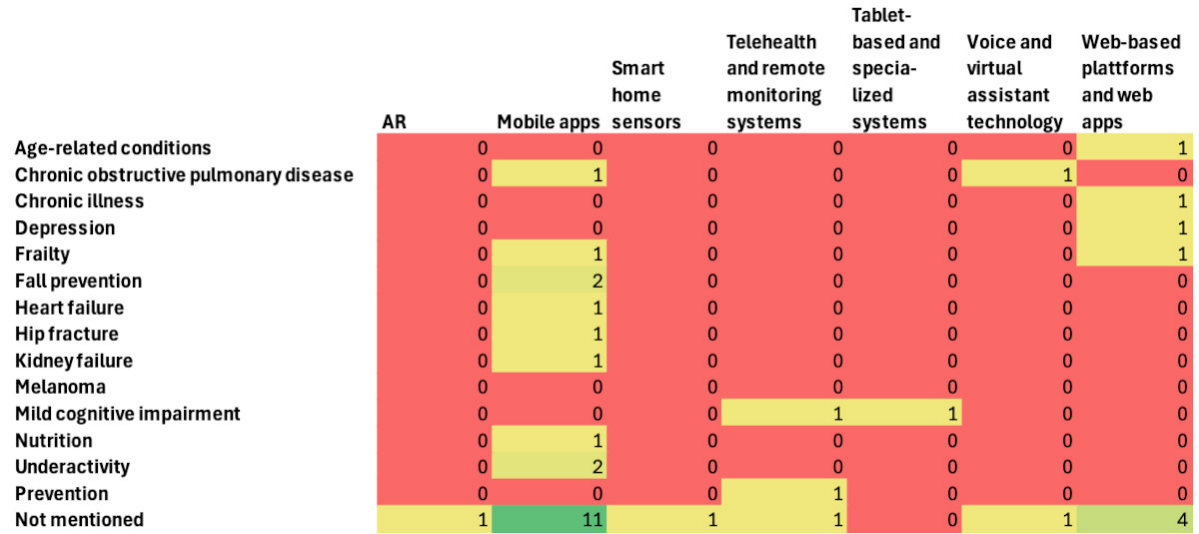
Gap analysis of diseases and technologies. AR: augmented reality.

Figure 2 shows the gap analysis between medical conditions and technologies. Mobile apps dominate, followed by web-based platforms. Tablet-based and specialized systems, voice and virtual assistants, smart home sensors and telehealth or telemonitoring, and augmented reality technologies are underrepresented across all health domains.

**Technical platforms on which the digital health interventions (DHIs) were implemented.**

- Mobile apps [30,31,34,35,37,39-43,45,46,49,51-53,55,57,60,64,65].
- Web-based platforms and web apps [25,28,29,32,59,66].
- Voice and virtual assistant technology [27,36].
- Telehealth and remote monitoring systems [26,42,44,48].
- Tablet-based and specialized systems [33,38,47,49,56,59].

Further, 28 papers did not provide information on the parties involved in the development of the DHIs (the DHIs’ primary users). Other groups were involved in DHI development, including (1) family and informal caregivers [42,47], (2) health care professionals (clinicians, geriatricians, cardiologists, nurses, physiotherapists, dieticians, occupational therapists, social workers, and mental health professionals) [50], (3) design and development teams (app developers, software engineers, UX researchers, and service designers) [28,31,42], (4) research and academics (interdisciplinary teams including sport scientists, sociologists, and university researchers) [40,43,56], and (5) other community stakeholders (community care professionals, community agencies, and aging service providers) [40,48,58]

The studies included in this scoping review used a variety of participatory and user-centered design methods to involve older adults in the design, development, and evaluation of DHI. Interviews and questionnaires, usability testing, focus groups, cocreation and participatory workshops, observational methods, mixed methods, and iterative feedback loops were featured in at least one study.

Semistructured and structured interviews [23,28-31,38-40,42-45,49,59,60,63,65,66] were used to elicit user requirements, explore experiences, and assess system usability. Structured questionnaires were also used to collect quantitative data on usability [56], user confidence [41], system acceptance [37,47], or user sentiment and requirement specification [55]. Natural language processing and sentiment analysis were used to further analyze the interview data [64].

Usability testing was conducted in various formats, including (1) think-aloud protocols during interaction with prototypes [31,42,66]; (2) task-based evaluations with mock-ups [55]; (3) structured usability surveys [26,37,49,53,58]; and (4) feasibility and acceptability tests to assess system functionality, ease of use, and user satisfaction, often occurring iteratively across different development stages [41,47,57].

Focus groups were used to gather group-level perspectives on needs, expectations, and experiences [30,31,34]. These sessions often informed requirements gathering and usability evaluation. Focus groups were sometimes combined with other methods, such as interviews or surveys, to gain a comprehensive understanding of user input [38-40,52,55].

Cocreational and participatory workshops were used to involve older adults in the design process, including (1) hands-on interaction with prototypes [29,31]; (2) group discussions and design exercises [32,38]; (3) interdisciplinary input from researchers, clinicians, and engineers [33,40]; and (4) community-based workshops and symposia [56].

Observational methods, often conducted in home or community settings, provided real-time insights into how older adults interacted with digital tools [36,39,66]. Interviews, scenario-based exercises, and mixed methods combining qualitative and quantitative techniques [49,50,56] enabled comprehensive evaluation through iterative cycles of design, testing, and refinement. Feedback was gathered through surveys, interviews, usability testing, and cocreation sessions.

### Design Guidelines and Frameworks for Inclusive Design for Older Adults

A total of 26 papers referred to user-centered design frameworks, digital and usability frameworks, health and behavioral science frameworks, cocreation guidelines and processes, health-specific and inclusive design guidelines, and study-specific conceptual models used in DHI design and development. Most of the frameworks were mentioned only in one of the papers, as shown in Textbox 4.

**Frameworks and design guidelines used in inclusive design implementation.**

**User-centered and human-centered design**

- User-centered design framework [50,54]
- Human-centered design approach [39,40,56]
- User-centric design principles [45,55] and design thinking [43]

**Digital and usability frameworks**

- Optimized user experience Honeycomb Model by Morville [52]
- Optimized Honeycomb Model by Karagianni [28]
- READHY framework [32]
- Broderick et al framework for health literacy apps [28]
- Framework for evaluating mHealth tools for older patients [37]
- Principles of web design for the older adults [44]
- Use of Technology to Engage in Adaptation by Older Adults or Those With Low or Limited Literacy (USABILITY) framework [33]
- Mobile App Rating Scale [45], System Usability Scale [52,53], and User Experience Questionnaire [43,52]

**Health and behavioral science frameworks**

- Medical Research Council framework for developing and evaluating complex interventions [46]
- Intervention Mapping Protocol by Bartholomew [41]
- Information-Motivation-Behavioral Skills Model for patient engagement [41]
- Behavior change taxonomy by Michie [46]
- Technology Acceptance Model [46]
- User acceptance and behavioral analysis models [47]
- Structural Model of Actors [48]
- Behavior Change Wheel (BCW) [52]
- Fogg behavior model [54]

**Cocreation guidelines and processes**

- PRODUCES + cocreation guideline [29]
- Co-design and participatory frameworks [33,38,54]

**Health-specific and inclusive design guidelines**

- World Health Organization’s Integrated Care for Older People framework [39]
- Inclusive design guidelines [34]
- User-sensitive inclusive design approach [49]

**Study-specific conceptual models**

- Study-developed conceptual frameworks [26]
- Accessibility Checklist for User Experience Designers, informed by Web Content Accessibility guidelines, Google, Princeton, and Team Treehouse, adapted for cognitive and visual impairments [28]

**Figure 3:**
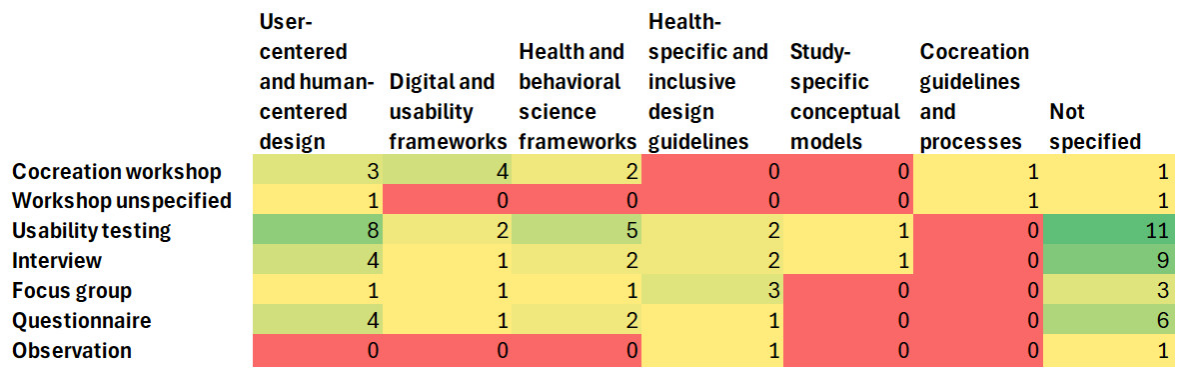
Gap map analysis of methodological approaches and frameworks.

A gap analysis of methodological approaches and frameworks (Figure 3) shows that, although usability testing is often guided by user- and human-centered design principles, it is frequently conducted without reference to any specific framework. Occasionally, usability testing is integrated with health or behavioral frameworks. Cocreation guidelines are referenced only when cocreation methods are explicitly used, and even then, inconsistently. Digital and usability frameworks are usually used during cocreation workshops. Notable gaps include the limited application of user- and human-centered design frameworks in focus groups and the rare integration of digital or usability frameworks with usability testing. Health-specific and inclusive design guidelines are occasionally used alongside focus groups, interviews, and usability testing, but not in workshop settings. Overall, frameworks are rarely mentioned in the included papers (“not specified”).

### Categories of Design Elements (Design Guidelines)

**Figure 4:**
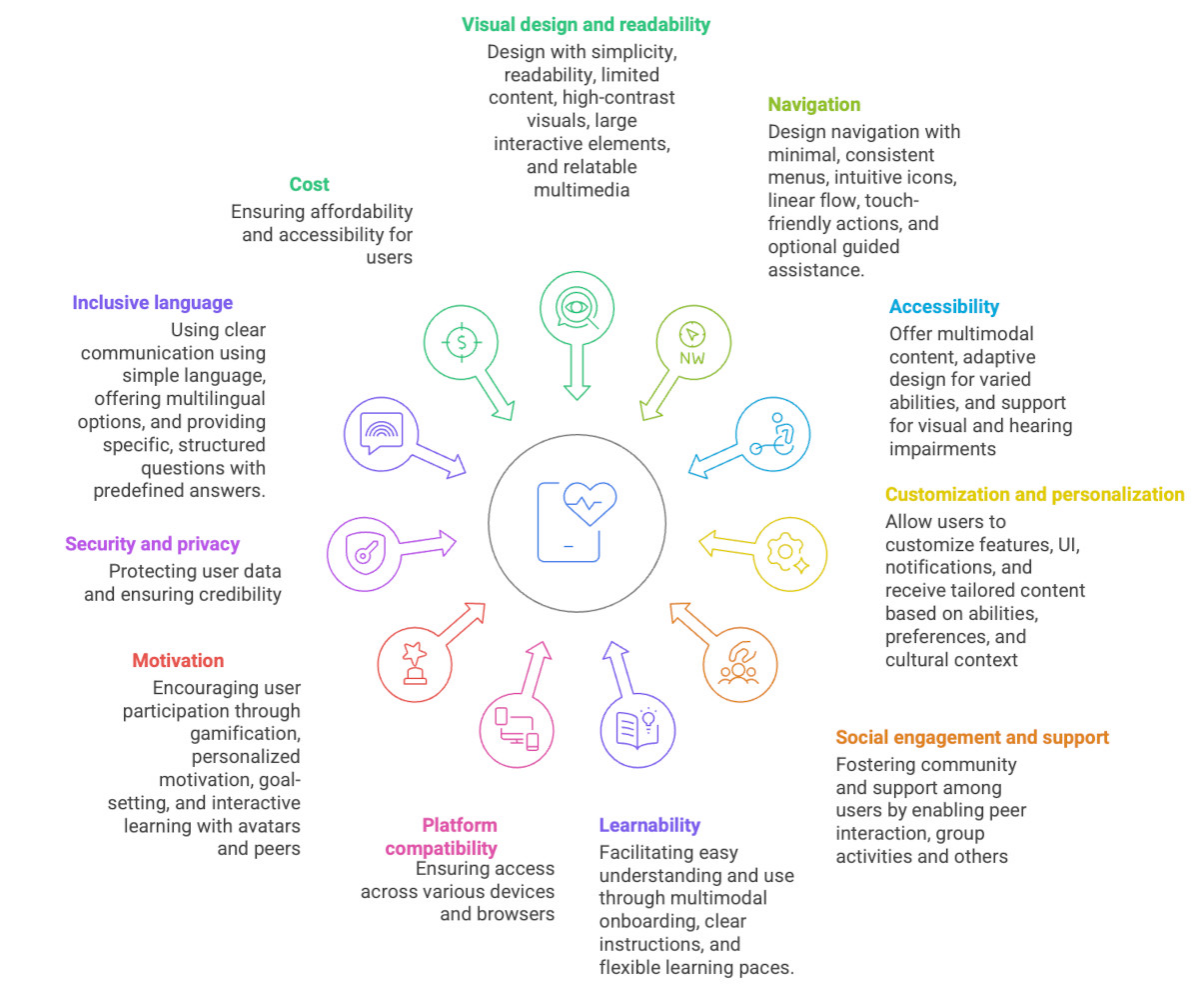
Eleven heuristics emerged from the literature analysis: visual design and readability, navigation, accessibility, customization and personalization, social engagement and support, learnability, platform compatibility, motivation, security and privacy, inclusive language, and costs. UI: user interface.

Eleven categories of design elements were identified in the included papers: visual design and readability, navigation, accessibility, customization and personalization, social engagement and support, learnability and educational content, multiplatforms and device compatibility, motivation, feedback and user engagement, security and privacy, and inclusive language and costs (Figure 4). Textbox 5 provides more details on the recommended design elements per design aspect. Figure 4 summarizes them in a visual manner.

**Inclusive design aspects and recommended elements to achieve inclusive design of digital health interventions (DHIs).**

**Visual design and readability**

- Use simple user interface design [53].
- Apply large-sized fonts [26,30,33,43,46,49,53] (eg, Helvetica [28]).
- Offer users the ability to increase font sizes [26,34,37,44,54].
- Implement large-size buttons [28,43].
- Ensure high-contrast between background and text [28,33,37,53].
- Use vivid colors [34,50].
- Present a limited number of options and on-screen information [28,38].
- Include photo and video material, especially featuring real older adults [28,29,31,40,49,51].
- Avoid pictures with family as they could be seen as discriminating [38].
- Use less text and more graphic elements (eg, pictures or animations) [39,42,53].

**Navigation**

- Use minimalist and simplified menus with self-explanatory icons [34,37].
- Use linear navigation structure and minimize user freedom to avoid cognitive overload [26,40].
- Support scroll-down functions and single-click options within the navigation [28,44].
- Use a program guide (eg, animated character or avatar) to assist navigation [29].
- Use large buttons placed consistently across all screens [37,40,53].

**Accessibility**

- **General accessibility recommendations**
  Provide multimodal content (in the form of videos, text, and audio) to address diverse needs [30,43,47].
  Enable voice interaction and touchless use [27,42].
  Design for different cognitive abilities [31].
  Tailor the app to the user’s age and impairments and avoid “one size fits all” DHI [45].
  Provide touch pens for users who have difficulties with touch screen [49].
- **Support for visual impairment**
  Provide voice assistants, reminder sounds, and voiceovers [29,30].
  Ensure compatibility with screen readers [34,37].
  Enable zoom functionality and screen magnification [33].
  Include extended text features to support future vision loss [28].
- **Support for hearing impairment**
  Offer instructions in written form alongside video material [31].

**Customization and personalization**

- Allow users to select preferred app features and content visibility [26,29].
- Enable customization of notification timings [35].
- Enable customization of user interface elements (eg, font sizes) [26,34,37,44].
- Provide tailored exercise programs and instructions based on individual physical abilities [30].
- Recommend activities and social opportunities based on user interests and state (eg, social groups and motivation) [36,39].
- Make culturally appropriate suggestions [39,53].

**Social engagement and support**

- Enable networking with other users to foster social and emotional support (eg, allow users to create social support groups using a Facebook-like interface; enable friend search by criteria such as name, hobbies, and health conditions) [26].
- Facilitate social engagement by recommending peer groups with similar interests [36,51].
- Encourage physical activity through group-based or peer-supported exercise functions [43,51].
- Support human-like interaction via conversation-based systems (eg, Alexa) to enhance human-like components of the DHI [27,52].
- Allow sharing of user data with caregivers and health care professionals [32].
- Include pop-up reminders or calendar functions for social events and activities [32].

**Learnability and educational content**

- Include an onboarding tutorial with multimodal content (eg, video, audio, and text) [32].
- Provide clear instructions on the purpose and benefit of specific exercises [33].
- Offer comprehensive educational materials that promote general well-being [35,52].
- Send random educational text messages with tips on self-care, diet, medication, and physical activity [41].
- Include videos for exercise demonstration [35,49].
- Ensure adjustable learning paces [39].
- Provide information and content from trusted sources (eg, World Health Organization and American Heart Failure Society) to ensure safety and credibility [30,41].

**Multiplatform and device compatibility**

- Enable access through all common browsers [26,28].
- Make the DHI suitable for PC, smartphone, and tablet [25,27].
- Avoid need for installation [27].

**Motivation, feedback, and user engagement**

- Integrate gamification elements such as scoring, progress tracking, and badges, which are shareable with other users [52].
- Send tailored daily motivational messages and reminders via push notifications [41,45].
- Enable interaction with avatars or peers during the learning process [39,50].
- Enable a goal-setting function and progress tracking [39,52].

**Security and privacy**

- Resist on requirement for password and username if possible [27].
- Emergency SOS (Save our Souls) function needs to be integrated [37].

**Inclusive language**

- Use simple and plain language [27,45].
- Enable option for selecting different languages [39,54].
- Use clear, specific questions with previously defined response options [40].
- Provide easy-to-understand, clear messages [29].
- Tailor messages to the user (eg, by mentioning the user’s name or the general practitioner’s name) [27].
- Use empathetic messages for communication of negative results [27].

**Costs**

- Ensure cost coverage of the DHI by the insurance [28].
- Provide DHIs free of cost for user [27].

### Barriers and Facilitators to the Adoption of DHI

The papers reviewed mentioned several barriers to the effective adoption and sustained use of DHIs by older adults. These barriers were grouped into the following thematic categories: technical barriers; usability and design challenges; cognitive, physical, and sensory limitations; psychological and motivational barriers; social and cultural barriers; systemic and structural barriers; and engagement and feedback-related issues.

Older adults encountered several technical barriers that limited their engagement with digital health tools. First, of device compatibility and access, some lacked access to adequate devices (eg, those capable of videoconferencing [26,27] or voice interaction). Others found smartphones and tablets incompatible or difficult to use due to user interface or UX issues [51]. Second, of connectivity and power, poor network connections, power instability, and internet access issues were common, especially in rural or underserved areas [26]. Third, of software issues, inaccurate voice recognition, unresponsive apps, software bugs, and unintuitive interfaces caused frustration and dropouts [26]. Finally, of touchscreen and input difficulties, challenges with touch screens, scroll menus, and small icons were especially problematic for those with arthritis or limited finger dexterity [34].

Usability and design challenges resulted from complex user interfaces (eg, unclear labeling and confusing navigation paths) [34]. Logging in, entering passwords, registering accounts, and verifying identities were commonly cited barriers. Lack of tailored content, unclear task instructions, and irrelevant app features diminished user engagement. Font sizes, colors, and interface elements often failed to meet accessibility standards for aging populations [42].

Age-related health issues, such as memory issues, difficulties in following instructions, or inability to achieve basic digital literacy [46], hampered DHI use. Sensory impairments such as poor vision, hearing loss, or reduced motor function made interaction difficult [38], as did physical impairments such as holding devices, prolonged screen time, or discomfort with extended DHI use.

Some papers described varying degrees of resistance toward adopting new technology among older adults. Fear of making mistakes, not trusting the technology, and worrying about fraud contributed to the reluctance [42]. Some older adults did not see the added value of DHIs, especially when alternatives such as caregiver support were available. Even with manuals and support, older adults were sometimes unwilling to learn due to perceived difficulty.

Social and cultural barriers also played a significant role in DHI acceptance, as well as in older adults’ involvement in design and development. Stereotypes that portray older adults as incapable [35], language barriers, and culturally irrelevant design affecting usability and confidence discouraged older adults’ involvement in DHI design and development.

At the organizational and infrastructure level, affordability of devices, subscriptions, or internet plans was a major concern for adoption [37]. Lack of professional prescribing of digital tools, poor promotion of assistive technologies, and limited access to services were cited as barriers to uptake. Privacy concerns, unclear ownership of personal health data, and lack of regulatory oversight contribute to limited trust in the DHIs [36].

The lack of real-time response and human support reduced the perceived value of the technology. Missing or poorly designed motivational elements, infrequent or poorly timed reminders, intrusive voice messages, and irrelevant prompts hindered habit formation [48]. Without habit formation, the older adults simply forgot to use the system.

The aspects that were extracted as facilitators represent the opposite of the barriers and concern individual factors, technological design factors, and social and behavioral support factors. They can be grouped into 6 categories and are summarized in Textbox 6.

**Facilitators of digital health intervention adoption among older adults with reported concrete aspects.**

**Personal motivation and attitudes**

- Willingness to learn and adopt technology [36].
- Desire for independence and self-care [36].

**Personalization and relevance**

- Customization options (eg, select the data to track) [47].
- Tailored reminders and messages [25].
- Cultural and language appropriateness (eg, clear language and accessible instructions) [45,54].

**Usability and accessibility**

- Ease of use and intuitive design [44].
- Adaptations for sensory or cognitive limitations [30].
- Visualizations and progress indicators (eg, graphs, status bars, and visual cues) [36].
- Supportive onboarding (eg, tutorials, in-app guides, and help from coaches) [44].

**Functionalities and health benefits**

- Data sharing with health professionals [46].
- Tools built on reputable sources (eg, World Health Organization guidelines) [39].
- Minimal data collection [48].

**Social support and interaction**

- Social inclusion features (eg, communication with peers or caregivers) [45].
- Human-like or conversational interfaces [26].
- Community and peer engagement (eg, group exercise scheduling and community calendars) [43,49].

**Engagement strategies**

- Gamification and interactive elements [45].
- Reminders and motivation boosters [46].
- Clear task instructions.
- Enjoyable interfaces [43].

## Discussion

### Definition of Inclusive Design

A primary objective of this work was to identify a set of principles for the inclusive design of DHIs for use by adults aged 60 years and older. Only 3 papers explicitly defined inclusive design, highlighting conceptual inconsistencies across the literature. Khamaj and Ali [37] emphasized accessibility and flexibility for users with mobility or dexterity challenges, while Wikberg-Nilsson et al [38] framed it as a design responsibility to include users with diverse needs. Chopvitayakun et al [53] introduced “user-sensitive inclusive design,” extending user-centered design to older adults and people with disabilities. To integrate these views, we adapt the definition presented in the introduction accordingly: inclusive design is an approach that aims to accommodate the needs of a broad spectrum of users by proactively considering variations in physical, cognitive, and social capabilities, as well as factors such as socioeconomic status, gender, age, ethnicity, and language diversity.

### Comparison to Standard Usability Heuristics and Principles

A comparison of the 11 derived design elements (design guidelines) with established frameworks, such as Nielsen’s usability heuristics and the Universal Design Principles (Table 2), reveals areas of alignment as well as extensions relevant to DHIs for aging populations. Our guidelines share fundamental aspects with existing frameworks, such as visual clarity, navigational consistency, and feedback mechanisms, all of which support usability and accessibility. However, they also encompass psychosocial and contextual dimensions that are critical for older adults, extending beyond traditional usability. Specifically, the inclusion of social engagement, motivational design, and cost considerations introduces equity and sustainability aspects that are underrepresented in the classical usability and accessibility models. Similarly, features such as personalization, multiplatform compatibility, and inclusive language adapt established principles to the heterogeneity of older users' abilities, preferences, and resources. This synthesis shows that, while established heuristics and guidelines provide a baseline for functional usability heuristics, inclusive design specifically targeting older adults places usability within a broader framework of social participation, trust, and empowerment—key factors in sustaining engagement and efficacy in DHIs for older adults.

**Table 2:** Comparison of our set of inclusive design guidelines with Nielsen heuristics and universal design principles.

Guidelines for DHIs^a^ for older adults	Nielsen heuristics [67]	Universal design principles [68]
Visual design and readability	Aesthetic and minimalist design, and visibility of system status	Perceptible information, and simple and intuitive use
Navigation	User control and freedom, and consistency and standards	Simple and intuitive use, and tolerance for error
Accessibility	Error prevention; and help users recognize, diagnose, and recover from errors	Equitable use and perceptible information
Customization and personalization	Flexibility and efficiency of use (partial match)	Flexibility in use
Social engagement and support	Not directly covered	Equitable use (partial match)
Learnability and educational content	Help and documentation, and the match between the system and the real world	Simple and intuitive use
Multiplatform and device compatibility	Consistency and standards	Equitable use and flexibility in use
Motivation, feedback, and user engagement	Visibility of system status and recognition rather than recall	Perceptible information and tolerance for error
Security and privacy	Error prevention (partial match)	Equitable use
Inclusive language	Match between system and real world (partial match)	Simple and intuitive use
Costs	Not directly covered	Equitable use

^a^DHI: digital health intervention.

### Reflections on Results

This scoping review synthesized evidence from 40 studies on inclusive design of DHI for older adults, revealing both progress and persistent gaps in this emerging field. Three key findings merit particular attention: (1) the recent growth in research activity suggests increasing recognition of the importance of inclusive design for aging populations; (2) despite this growth, substantial evidence gaps persist across DHI types, health domains, and methodological approaches; and (3) there remains a concerning disconnect between stated inclusive design objectives and actual implementation of inclusive design principles.

The predominance of mobile apps is both logical and limiting. While smartphones offer portability, sensors, and always-available functionality suitable for health monitoring and behavior change interventions, the mobile-first approach may inadvertently exclude older adults with limited smartphone access or a preference for larger screens. The minimal representation of web platforms is particularly concerning, given that many older adults report greater comfort with computer-based interfaces and that websites can be more easily accessed across devices without requiring app installation skills.

The research studies included in this analysis were developed to facilitate DHI use among people with a wide range of common physical disabilities, including vision impairments, hearing impairments, and sensory decline, as well as mild cognitive impairment. In some instances, co-design participants had lived experience of a particular health condition or physical limitation, while in other initiatives, a cohort of older adults with no specific pre-existing conditions provided user feedback. Future research could involve older adults with age-specific disabilities in more depth to cover these aspects in DHI design to a larger extent.

Furthermore, the initiatives described in the studies were designed to fulfill common health-related goals, for example, prevent falls, increase physical activity, and address depression. As these goals are common to older adults all over the world, it is possible to infer that the principles identified can be generalized to many locations and clinical settings. The inclusion of individuals with a diverse range of functional challenges suggests that the learnings from this study will be generally applicable to community-dwelling older adults. However, our analysis also indicates that inclusive design has not been applied to certain age-related medical conditions within the reviewed literature, notably diabetes, pain management, medication adherence, and social isolation.

The studies included in this analysis involved the co-design of 5 types of DHIs: mobile apps (20 studies), web-based platforms and web apps (6 studies), tablet-based and specialized systems (8 studies), telehealth and remote monitoring systems (4 studies), and voice and virtual assistants (2 studies), as noted in Table 2. As a result, all major categories of DHIs currently in use as nonexperimental tools are represented in the analysis. The preponderance of work involving mHealth apps is unsurprising given the maturity and broad acceptance of mobile technology and its usability across community and care settings. The inclusion of work on specialized systems, telehealth applications, and remote monitoring systems broadened the experience captured beyond the mHealth and tablet device sector, ensuring that this analysis is relevant beyond mHealth design. Research on inclusive design considering other technologies is needed (eg, voice and virtual assistants, tablet-based systems, smart home sensors, and wearables).

Similarly, the design heuristics identified in the studies included in the preset analysis (Textbox 4) encompass a broad range of design aspects grounded in human-centered design, web accessibility guidelines, interface usability, digital UX, and the biology of aging. Distributed among 11 design aspects, the recommended inclusive design elements provide broad, deep guidance for design and co-design of DHIs to be used by older adults. Some elements (eg, apply large-sized fonts and design for different cognitive abilities) are so well-understood as to seem like common sense, while other elements (eg, enable interaction with avatars during the learning process) historically have been less used. This collection of design aspects forms a repository of best practices for design for older adults as well as for other groups (eg, people with disabilities and digital interactions in patients’ nonpreferred languages) and points out opportunities for additional research into inclusive design.

A particular value of the present work is its identification of design elements that facilitate the adoption of DHIs by older adults. The most carefully planned development effort is of no consequence if the intended population is unable or unwilling to integrate the DHI into daily life in real-world settings. The 6 categories of facilitators—personal motivation and attitudes, usability and accessibility, personalization and relevance, social support and interaction, functionalities and health benefits, and engagement strategies—offer a comprehensive view of personal and situational factors underlying DHI adoption. This analysis may be particularly valuable in resource-constrained environments in which inclusive design efforts must be tightly focused on filling specific gaps.

Personalization emerged as both a frequent design principle and a top facilitator to adoption, yet this creates tension with the principle of simplicity. Offering personalization requires users to understand what can be customized, navigate to customization settings, make informed decisions about preferences, and potentially manage increased complexity. Balancing personalization with simplicity requires sophisticated design that provides sensible defaults, progressive disclosure of advanced features, and clear guidance—challenges that few studies explicitly addressed so far and remain open for future studies.

The co-design efforts included in the present analysis were developed in response to perceived community needs and, in many cases, were designed around available resources within the given setting. This circumstance created significant variation among the study designs and resulted in an abundance of real-world experience that supports the generalizability of the learnings. The variability of the older adult co-design participants, the methods used to elicit feedback, and the range of health promotion objectives among the individual studies surfaced neither universal design principles for the older adult population nor effective co-design strategies for this group. Rather, this study identifies a range of ways to co-design DHIs and provides insight into the types of learning that may be realized through these approaches.

While some studies adapted existing frameworks or developed new ones specifically for older adults, the overall application of design frameworks remains limited. This may be due to the scarcity of frameworks explicitly tailored to DHIs for this population, as well as the persistent gap between theoretical models and their practical implementation. Existing frameworks frequently emphasize specific dimensions, such as usability or UX, rather than addressing the broader context of use. However, the design of DHIs for older adults requires consideration of the entire ecosystem into which these technologies are integrated, including all relevant contextual and interactional factors. The following practical implications highlight key aspects that warrant attention in future research and development efforts.

### Practical Implications

The resulting guidelines highlight that the inclusive design of DHI extends beyond traditional considerations of usability and user interface design. Instead, inclusive design involves addressing a multitude of dimensions that must be deliberately integrated into the design process. This underscores the growing importance of participatory design methodologies, which ensure comprehensive coverage of diverse factors influencing user engagement and the effectiveness of DHIs.

Additionally, organizational and infrastructural considerations must be rigorously documented and incorporated into design decisions. As individuals age, they often experience increased dependency and reduced flexibility in adapting to new technologies. Therefore, DHIs should proactively incorporate mechanisms such as peer support or interactions facilitated by health care professionals. These interpersonal components are essential for building trust, demonstrating the practical usefulness of the technology, and providing immediate assistance to overcome technical obstacles.

Adopting a participatory design approach [69] ensures that diverse perspectives and experiences from the target demographic are systematically gathered and used. Our proposed design guidelines offer a set of practical best practices and recommendations. However, it is important to note that designers do not need to apply each element of the guideline universally. Instead, the design must avoid a “one size fits all” philosophy, emphasizing customization and alignment with the specific needs and preferences of each unique user group targeted by the DHI.

The findings of this work have important implications for the practice of health and health care more broadly. The need to mitigate older adults’ isolation and loneliness was highlighted during the COVID-19 pandemic, when individuals worldwide were expected to remain at home for weeks to months, curtailing all routine social activity and in-person interactions with others [70]. In nonpandemic times, the common life experiences of loss of mobility, loss of vision and hearing, lack of transportation, insufficient financial resources, and others prevent older adults from accessing in-person care, necessitating eHealth solutions [71]. The range and variety of DHIs developed through co-design with older adults and the ability to create rich digital experiences with minimal reliance on validated design frameworks observed in the analysis suggest that co-design supports flexibility and tailoring of DHIs to local populations and conditions. This opportunity for customization will be key to the success of health service delivery for older adults and improved health outcomes as countries worldwide seek to address the needs of aging populations. The increase in publications on inclusive design of DHI for older adults after the COVID-19 pandemic indicates that researchers are starting to think about these aspects. However, much more research is needed, as the identified gaps show.

Furthermore, this work underscores the need for affordable, reliable internet access as well as simple, functional patient portals built for people with a range of abilities. The need for broadband access has been well-understood for years [72], but remains out of reach for many [73,74]. The digital divide is often conceptualized as a technological problem, but acceptability, adequacy, and affordability dimensions play a role as well [75]. People who live at a distance from health care facilities and must access some of their care or health-promoting services online benefit from co-design of DHIs because co-design facilitates accounting for environmental, technological, and cultural barriers from the start.

### Limitations

The included papers did not provide all the information that was of interest. Specifically, the development and design processes were often not well described; for example, 28 papers did not provide details on who was involved in the development process. Some papers were excluded because the digital solution was not or insufficiently described. Consequently, bias might have been added to our results. To address this limitation, we conducted in parallel expert interviews and a participatory workshop with older adults, and we will integrate the results into the set of guidelines presented here.

In addition, the definition of the term older adult was not used in a consistent manner throughout the different papers included. Sometimes, papers described the solution for older adults, but younger adults, for example, those aged 20 years, were included in the study. Those papers were excluded from our study.

Another limitation of this scoping review is that the review protocol was not registered in advance, which may reduce transparency and increase the potential for unintentional bias in the review process. To address this limitation, all three authors critically reflected on the different steps. The included papers were not assessed systematically regarding their methodological quality using tools. For this reason, we are unable to distinguish limitations of the design of DHI from reporting gaps and study quality issues of the included papers.

The guidelines presented in this paper result from a literature search. Thus, the DHI considered might be research prototypes, probably not covering the practical perspective completely.

### Conclusions

Our findings suggest that inclusive DHI design for older adults should be considered a multidimensional process integrating psychosocial and contextual factors alongside usability and accessibility. Emphasizing social engagement, motivational features, and affordability enables designers to address equity and sustainability gaps that classical usability approaches do not adequately capture.

With these results, we contribute to our 3-phase development process of inclusive design heuristics of DHI for older adults. We proposed an initial set of inclusive design guidelines, which will be integrated with the information collected from other sources, such as expert interviews and participatory workshops with older adults, into a set of heuristics as a next step. Once this has been done, the resulting list of heuristics needs to be validated and improved. Future work could develop an evaluative checklist using the final set of heuristics that can be used in the co-design sessions of DHI with older adults or within usability testing and heuristic evaluations.

## Appendix

Multimedia Appendix 1 PRISMA-ScR checklist.

Multimedia Appendix 2 PRISMA-S checklist.

Multimedia Appendix 3 Detailed search strategy for each database.

Multimedia Appendix 4 Data extraction table with reasons for exclusion.

## Abbreviations

DHI: digital health intervention
Got-IT: Guidelines on Target Assessment for Innovative Therapeutics
mHealth: mobile health
PRISMA: Preferred Reporting Items for Systematic Reviews and Meta-Analyses
PRISMA-S: Preferred Reporting Items for Systematic Reviews and Meta-Analyses literature search extension
PRISMA-ScR: Preferred Reporting Items for Systematic Reviews and Meta-Analyses extension for Scoping Reviews
UX: user experience

## Data Availability

The data extraction table is provided as Multimedia Appendix 2 together with the paper.
