# Supplementary material for: Toward Inclusive Design Heuristics for Digital Health Interventions for the Aging Population: Scoping Review
Source: J Med Internet Res. 2025 Dec 22;27:e79449. doi: 10.2196/79449 (PMC12770925; doi:10.2196/79449)
Supplement: Multimedia Appendix 3 [file jmir_v27i1e79449_app3.docx]

Database 1: IEEE Xplore

**Applied search strategy:**

("digital health" OR "eHealth" OR "mHealth" OR "telemedicine" OR "telehealth" OR "digital healthcare tools" OR "digital therapeutics" OR "mobile health apps" OR "online health platforms" OR "wearables")

AND

("aging population" OR "older adults" OR "seniors" OR "aged individuals" OR "older persons" OR "geriatric population" OR "aging individuals" OR "aging adults" OR "senior citizens" OR "elderly population")

AND

("inclusive" OR "accessible" OR "equitable" OR "universal design" OR "user-friendly" OR "culturally sensitive" OR "inclusive design" OR "barrier-free" OR “non-biased” OR "non-discriminatory" OR "diverse" OR "adaptive" OR "tailored for all")

Database 2: Scopus

**Applied search strategy:**

("digital health" OR "eHealth" OR "mHealth" OR "telemedicine" OR "telehealth" OR "digital healthcare tools" OR "digital therapeutics" OR "mobile health apps" OR "online health platforms" OR "wearables")

AND

("aging population" OR "older adults" OR "seniors" OR "aged individuals" OR "older persons" OR "geriatric population" OR "aging individuals" OR "aging adults" OR "senior citizens" OR "elderly population")

AND

("inclusive" OR "accessible" OR "equitable" OR "universal design" OR "user-friendly" OR "culturally sensitive" OR "inclusive design" OR "barrier-free" OR “non-biased” OR "non-discriminatory" OR "diverse" OR "adaptive" OR "tailored for all")

Database 3: PubMed

**Applied search strategy:**

("digital health" OR "eHealth" OR "mHealth" OR "telemedicine" OR "telehealth" OR "digital healthcare tools" OR "digital therapeutics" OR "mobile health apps" OR "online health platforms" OR "wearables")

AND

("aging population" OR "older adults" OR "seniors" OR "aged individuals" OR "older persons" OR "geriatric population" OR "aging individuals" OR "aging adults" OR "senior citizens" OR "elderly population")

AND

("inclusive" OR "accessible" OR "equitable" OR "universal design" OR "user-friendly" OR "culturally sensitive" OR "inclusive design" OR "barrier-free" OR “non-biased” OR "non-discriminatory" OR "diverse" OR "adaptive" OR "tailored for all")
